# Augmenting Circadian Biology Research With Data Science

**DOI:** 10.1177/07487304241310923

**Published:** 2025-01-29

**Authors:** Severine Soltani, Jamison H. Burks, Benjamin L. Smarr

**Affiliations:** * Bioinformatics and Systems Biology Graduate Program, University of California, San Diego, La Jolla, California; † Shiu Chien-Gene Lay Department of Bioengineering, University of California, San Diego, La Jolla, California; ‡ Halıcıoğlu Data Science Institute, University of California, San Diego, La Jolla, California

**Keywords:** data science methods, big data, time-series, computational biology, statistics

## Abstract

The nature of biological research is changing, driven by the emergence of big data, and new computational models to parse out the information therein. Traditional methods remain the core of biological research but are increasingly either augmented or sometimes replaced by emerging data science tools. This presents a profound opportunity for those circadian researchers interested in incorporating big data and related analyses into their plans. Here, we discuss the emergence of novel sources of big data that could be used to gain real-world insights into circadian biology. We further discuss technical considerations for the biologist interested in including data science approaches in their research. We conversely discuss the biological considerations for data scientists so that they can more easily identify the nuggets of biological rhythms insight that might too easily be lost through application of standard data science approaches done without an appreciation of the way biological rhythms shape the variance of complex data objects. Our hope is that this review will make bridging disciplines in both directions (biology to computational and vice versa) easier. There has never been such rapid growth of cheap, accessible, real-world research opportunities in biology as now; collaborations between biological experts and skilled data scientists have the potential to mine out new insights with transformative impact.

## Introduction: Biological rhythm insights are being mined from new data, but you need more than a pick and shovel to make it at the frontier

The study of biological rhythms has changed from a niche field into one that has real-world impacts across society. Broader audiences are recognizing the importance of biological rhythms in their own lives or fields. This successful growth is in part due to the fact that the world has itself gone through a transformation—it has become data rich. An investigation into biological rhythms used to require massive efforts to generate enough data to capture biological effects at multiple time points across a cycle. Today, many such experiments continue, as they should. However, new opportunities have emerged to complement these works. An overwhelming abundance of ambient data are generated primarily for purposes unrelated to circadian studies. However, with sufficient temporal resolution, researchers can dig into them to mine out insights about biological rhythms in the world. Examples include wearable device data from populations using personal sleep or fitness trackers (Smarr et al., 2020; Viswanath et al., 2024), social media activity (Roenneberg, 2017), livestock management (Aguirre et al., 2021), or urban infrastructure (Schirmer et al., 2019). The emergence of this new scientific method—mining for findings from mountains of ambient or already extant data about the world—is exciting, as it allows us to imagine seeing biological rhythms interacting with environmental inputs in ways (and at spatiotemporal scales) never before possible (and still perhaps well beyond the scope of what could be funded by a normal research proposal).

While many people recognize that biological rhythms are fundamental to the health and well-being of organisms and ecosystems, few actually make use of them by integrating them into their standard analyses. Many scientists, clinicians, or otherwise still do not by default make decisions that take into account the rhythmic structures within the materials they deal with (e.g., medicine does not by default look at the time of day as critical for interpreting measurements or administering medications; Albuquerque et al., 2021). The ubiquity of rhythms, on one hand, and the absence of optimization in most systems around these ubiquitous rhythms, on the other hand, mean that as data render the presence (or at least proxy traces) of these rhythms numerically accessible, they open opportunities for truly massive, transformative impacts in and across fields. The rhythms being optimized around are increasingly visible and tractable through these data, and so opportunities for improved optimizations abound and continue to expand.

This transformation into a data-rich world also means that many data types and sources are new to research communities, as they are new to everyone, and so deserve fresh consideration as to their value for research enterprises. Some now-digitized data sources make more obvious targets for biomedical research, such as medical records, county mortician logs, satellite imagery for weather, climate, and light at night. Some others may be less obviously useful in biomedical research because they appear (or are) less immediately related, as in computational telemetry data, traffic flow or collision records, and university learning management system (LMS) logs. Others might be less obvious because the analyses required to discern patterns were not previously accessible due to advances in automated analysis tasks, as in using large language models to assess changes in the frequency of textual content from social media across millions of users. In both cases, the data are generated by living systems (mostly humans, but not always, as in agricultural data or pest management data) and so if as a community we are right about the ubiquity of biological rhythms, these data ought to reflect or echo the rhythms of their creators. Even if the resulting rhythms’ residuals are noisy or weak, the abundance of data enables large-scale analyses that may reveal their existence with greater clarity than would be possible with smaller samples taken more directly. Especially when measuring humans in a modern societal context, there are so many other inputs that might shape an individual’s physiology and behavior that extracting trends from biological cycles will be very hard from any handful of individuals or samples, so that the presence of non-random, rhythmic influence may only become discretely measurable with measurements at scales of millions or more.

As the opportunity to find patterns from real-world data expands, by their nature they challenge the way most of us were trained to do science. For example, the use of parametric tests like the *t* test rest on the assumption that one has to idealize a distribution to mathematically compare it to another. By contrast, resampling from very large data can allow for data-specific probabilities of finding any given set of observations without any need for assumptions about what the Platonic ideal of the distribution should have been. The former evolved when a scientist would carry out calculations on a small set of data by hand. This is still useful in many cases and is the core of most scientific training in statistics in part due to cultural momentum; the latter offers a different and scalable alternative appropriate for science done on these newer, emergent sources of much larger datasets, where in the assumptions of the former approaches are likely to be inappropriate and misleading. Machine learning (ML) or signal processing are not part of the average biology curriculum, nor are the various concerns that arise from data management, cleaning, missingness, and so forth, but without some exposure to data science tools, it is hard to gain new biological insights from these new data-enabled opportunities.

That is not to say that every biologist or clinician should become a computer scientist. Far from it! Biological insights are critical to appropriate experimental design and analysis (deciding which data to acquire and what to look for) as well as for feature engineering (curating what an algorithm needs to see or know to efficiently make sense of the data; Futoma et al., 2020). And the truth that old ways may be inappropriate in a new context cuts both ways. Engineers and computer scientists have not traditionally had access to large sets of data from biological systems, but instead principally electrical and mechanical ones—engineered systems from which idealized distributions might well be expected. As a result, many attempts to apply engineering principles to algorithms for use on biological systems (Gianfrancesco et al., 2018; Ledford, 2019; Futoma et al., 2020) lead to biases and harm due to the same failure to fit the more complex structures created by data from biological systems.

Luckily, research is an increasingly collaborative enterprise (Jones et al., 2008; Jones, 2009). To make safe and meaningful headway in biological research with big data, it is increasingly important that biologists understand some of the tools used by data scientists and data engineers. In part so that they can make appropriate use of new techniques and data sources, and in part so that they can communicate with collaborators with computational but not biological training, to enable analytic designs that appropriately involve insights only available to experts with experience about biological systems. The circadian community is privileged to know that rhythms both exist and matter. Either as individual investigators or through collaboration with computational colleagues, the circadian community has a unique opportunity to transmute these myriad ambient data sources into sources of biological insight.

## Review Overview

In this review, we seek to make clear some Big Data opportunities specific to biological rhythms research. There is not room to deeply review every technique and caveat, as these describe whole fields. We intend only to make it easier for those interested to start their journeys and warn them of the common pitfalls to watch out for. We hope this will be of interest whether you want to become a “dry lab” researcher or simply want to be able to collaborate more directly with your computational colleagues. Specifically, we will give examples of new data sources in the hopes of stimulating those interested to dig for other such sources. We then cover why sources like this that continue to emerge could be promising resources for biological rhythms research. We will then discuss common needs, such as tools and toolboxes from commonly used programming languages, such as Python’s SciPy (Virtanen et al., 2020), and approaches such as parameterization of data for rhythms analysis, and how various forms of signal processing and modeling can support classical statistics. To support adoption of best practices along with these new tools, we will also discuss common concerns, including issues of incomplete or missing data, and touching on *p*-values in datasets where a 5% false discovery rate could still represent millions of relationships as well as using within-subject replicates to increase information despite challenging classical statistical notions of independence.

We constrain our review to materials related to what we believe describe less obvious resources. Many reviews exist about medical records, for example, and so we will treat such well-trod ground more lightly here, focusing on what might be new ground for more readers. What this review will not cover are specific implementations of ML or deep learning (DL) algorithms. We believe focusing on the data-driven parameterization of biological rhythms is more relevant than the application of artificial intelligence in and of itself for detecting structure. As researchers interested in biological rhythms, we know *a priori* that we want to extract information regarding cyclicity in longitudinal biological data. The features or the information extracted from such data can be used in ML algorithms if we hypothesize the model may surface differences between classes. However, the ML algorithms themselves may not inherently identify cyclicity. DL models would have to re-derive the rhythmicity or nonlinear dynamics we know are present in the observed signals. If the way we featurize data does indeed capture rhythmicity, we would then want to compare these parameterizations from cohorts of interest rather than assume a model can implicitly infer the cyclic structure. A full review could (and probably should) be written on the applications of ML and AI to biological rhythms data, but that will be most impactful for those already familiar with appropriate parameterization.

## New Data Sources

The emergence of digital devices and Internet infrastructure have led to many systems in the world generating data across time where those data did not exist historically. Many systems now generate data that capture processes in which either circadian rhythms play a role, or which impact the expression of biological rhythms. More directly, there exist numerous data sources from which one can search for and analyze rhythmic patterns related to biological systems. There are large datasets of human health data, many of which include longitudinal physiological data. Continuous monitoring via use of wireless devices can also be used to monitor non-human physiology in agricultural and ecological settings. For example, wireless sensors can be used to better understand and monitor risk factors that threaten livestock health; improvements in these realms have led to the advent of precision agriculture (Castro-Costa et al., 2015; Fontana et al., 2015; Neethirajan, 2017; Hong et al., 2020; Zaman and Dorin, 2023). Continuous monitoring may also be used to monitor the health of household pets (Brugarolas et al., 2016; Mekha and Osathanunkul, 2020). All of these datasets may provide time-series observations amenable to novel biological rhythms insights.

In contrast to these directly health-related data, the vast majority of modern datasets were not generated with biological research in mind. Most of them do not directly measure biological variables (blood, gene expression, etc.) either. These “non-biological” data can nevertheless reveal important information about circadian rhythms. For instance, the times at which students log into online LMSs does not directly measure their sleep or circadian rhythms, but it is dependent on their waking engagement with academic materials, which are in turn informed by the wake-sleep rhythms of each student (Smarr and Schirmer, 2018). These data can thereby serve as proxies for the desired signals of circadian biology. Soliciting enough funding to buy sleep measurement systems for thousands of students, and getting them to use these devices every night for months, would be hard to say the least. When those same students generate data just by being students, then there is a trade-off of abundance of data and directness of measurement. Abundant measurements are not globally better than smaller, but more direct, measurements (if that needs to be said); direct measurement will forever be valuable, and arguably most of circadian biology as a field has evolved to optimize for use of direct measurements. However, because data abundance is a relatively new phenomenon, there are fewer instances of using large, ambient (extant, generated for other reasons) datasets as tools with which to ask questions about the manifestations of biological rhythms in the real world. This novelty creates an opportunity for new research.

Many data types might fall within this category of “sources of proxy signals for biological rhythms.” In Table 1, we provide a non-exhaustive list of several datasets that are generally free to access by various means, drawing from both direct and indirect/proxy measurements, as well as environmental/contextual datasets. We hope that this table, along with a couple of examples that follow, gives an abstract template to the ways that various datasets can be put to the unintended purpose of serving biological rhythms analyses.

**Table 1.** Data sources (with cartoon example questions these could support).

Name	Accessibility	Notes	Example Questions
*Biological Datasets*			
PhysioNet (Goldberger et al., 2000)	Freely available/free credentialed access	Demographics; lifestyle; sensor data; physiological measurements; EHR; images	Does circadian stability of vital signs (e.g., heart rate, blood pressure) improve heart failure prediction?
UK Biobank (Sudlow et al., 2015)	Paid tiered access	Demographics; lifestyle; genomics	
EuroBioBank (Mora et al., 2015)	Contact researchers	Biological samples; rare diseases; genomics	Which genes and variants are associated with a proclivity toward shift work and less associated physical/mental burnout?
Estonian Biobank (Leitsalu et al., 2015)	Contact researchers; paid access	Demographics; lifestyle; genomics	
All of Us (All of Us Research Program Investigators et al., 2019)	Freely available; limited amount of free credit for analysis	Demographics; lifestyle; genomics; wearables; EHR	How do acute physical injuries impact activity levels, and is there a normative, injury-dependent, time-till-recovery before baseline activity rhythms are restored?
American Gut Project (McDonald et al., 2018)	Contact researchers	Microbiome	
*Ambient Datasets*			
PurpleAir	Limited free credit	Air pollution	Are there daily rhythms in specific pollutants, and does urban density flatten or amplify these rhythms?
MeteoStat (Lamprecht, 2024)	Freely available	Climate; weather	
Earthdata	Freely available	Geographic; climate; weather	Do nighttime light pollution levels (via satellite imagery) correlate with the prevalence of sleep disorders?
U.S. Traffic Volume Data	Freely available	Hourly traffic volume	
*Assorted Datasets*			
UCI Machine Learning Repository (Kelly et al., 2023)	Freely available	Assorted	Is there an association between time-of-day vaccination and subsequent antibody titer levels and infection rates?
UCR Time Series Classification Archive (Dau et al., 2018)	Freely available	Assorted	In what manner does the circadian pattern of social media posts change in response to positive versus negative valence news?
Kaggle	Freely available	Assorted	
data.world (Jacob and Ortiz, 2017)	Freely available	Assorted	
Papers with Code	Freely available	Assorted	How does chronotype and/or sleeping habits impact student performance?
Hugging Face (Lhoest et al., 2021)	Freely available	Assorted	
Dataset Search (Brickley et al., 2019)	Freely available	Assorted	What is the relationship between sleep-wake rhythm stability and depression over time, accounting for seasonal influences?
Data Planet	Freely available	Assorted	

As a first example, collaborators working with the Lincoln Park Zoo used trap camera data to model the level of brightness that inhibited animal movement through locations across Chicago at night. They then correlated these observations with the light level provided by the satellite images for those locations from Google Earth and extrapolated the corridors likely to be amenable to wild animals across the whole Chicago area. They found that a large proportion of the areas designated as wildlife corridors were in fact likely too bright to allow animals to make use of them (Schirmer et al., 2019). This project took pre-existing data and turned them into actionable policy suggestions for wildlife management based on circadian rhythms and environmental light pollution.

Somewhat differently, longitudinal monitoring of infrastructure data revealed a disproportionately higher frequency of traffic accidents in low-income and minority neighborhoods compared with more affluent areas (Dumbaugh et al., 2020). The insights afforded by these data can open up new avenues for research, such as investigating the effects of shift-work on the sleep patterns of minority populations, a factor known to contribute significantly to traffic accidents (Mujawar et al., 2021; Palm et al., 2023).

## Step 1: get data; step 2: . . .; step 3: publish

Thinking about questions one can ask with ambient, emergent data sources is fun. Implementing research to carry out those analyses often sounds easier than it ends up being. The challenge is often not that the scientific question has to be nuanced or complex. Instead, often reasonable analytic plans run into barriers due to the nature of the data themselves. Some of these problems arise prior to the researcher actually gathering data of interest. Problems such as tools for measurement and length of monitoring can have extended experimental consequences if not thoroughly considered before implementation. After data collection or extraction, many of these problems are not conceptually different from those one learns when practicing statistics, such as missing data and censoring, distributions that are not normal, and mediating factors underlying correlations. However, due to the size and complexity of many Big Data objects, certain best practices are the ropes, tents, and bear spray that are helpful to carry along with a shovel and pick when digging for gold in the wild data frontiers.

We discuss these best practices in the following sections. For the sake of applicability and consistency, we use an example of mouse core body temperature (CBT) from Smarr et al. (2016) to show applications of the methods discussed throughout this work. We selected a 3-day window of temperature sampled every minute and applied the algorithms discussed in subsections Considerations for Generating Data, Imputation, Noise, Outliers, and Data Analysis Techniques for Time-Series. It is worth noting that many of the issues discussed in the following sections are not unique only to Big Data analytics. We hope they may inform analyses broadly, but we include them specifically here because without these considerations, those to whom Big Data analyses are new are likely to fall into well-known traps and dead-ends. The material we summarize here is neither only those things to consider with Big Data nor all of the things one can consider with Big Data, but what we consider an essential foundation to confidently and purposefully marching off into the hills of Big Data with some expectation of striking new findings. To support confidence with these concepts, we make the data and Python code used to generate figures available in the Supplementary Materials.

### Considerations for Generating Data

#### Sampling Rate

**Figure 1.**
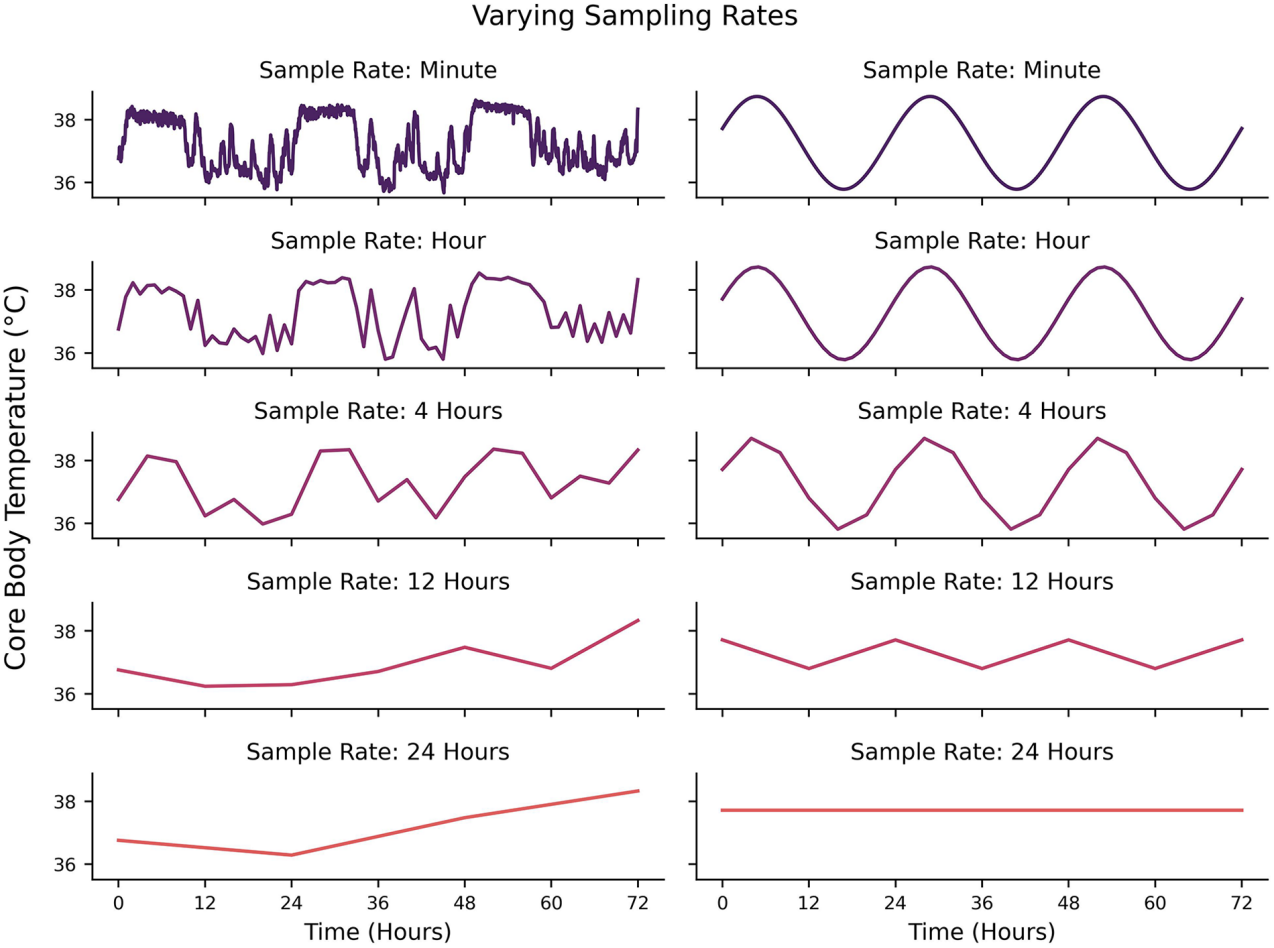
Left: Effects of different sampling rates on the same underlying signal from 1 min sampling (top) to 24 hr sampling (bottom). Right: Effects of different sampling rates on an idealized 24-hour periodicity from 1 min sampling (top) to 24 hr sampling (bottom).

Some technical considerations regarding data generation (or selection, in the case of preexisting datasets) should be made depending on the nature of the questions being asked. One of the most fundamental considerations is related to the highest frequency/lowest periodicity of interest (Shannon, 1949; Refinetti et al., 2007; Klerman et al., 2017). A signal that is hypothesized to have a periodicity of 24 h should be sampled *at least* every 12 h. Put generally, one should sample at a minimum frequency of twice the hypothesized frequency of their signal of interest (the Nyquist Rate). This ensures that if a cyclic structure exists, a researcher should at least be able to infer some rhythmicity in the data. However, this is a strict lower bound on the sampling rate and does not guarantee signal reconstruction either because the phase of measurements might fall away from the peaks and troughs of the signal (e.g., sampling all of the y=0 values from a sine wave leads to the reconstruction of a flat line, not a wave) or the signal might be non-stationary (meaning each repetition is not exactly the same, which is often the case in biological systems) (e.g., Figure 1). When dealing with nonstationary signals, it is no longer the period but the deviation of each period that should be considered when determining sample frequency (Refinetti, 2004). An intuitive example could be that detecting a 24-h rhythm in human sleep-wake states requires sampling at least every 12 h, whereas detecting differences in bed times across days might require sampling every few minutes. Another consideration is the length of time for which a signal is sampled. In the presence of noise, more samples are typically needed to confidently estimate signal using spectral analyses (Levine et al., 2002). Oversampling becomes more practical to perform when the signal-to-noise ratio decreases. For example, if a cyclic signal of interest does not have large magnitude relative to potential noise, more data will need to be acquired to increase the confidence in differentiating the signal’s cyclicity from variance caused by noise itself.

#### Sampling Length

If one is interested in the stability of rhythms within a dataset, then it may not suffice to only have enough data to test for the existence of cyclicity. It is necessary to have more longitudinal data in order to quantify the change in some features that describe cycle stability over time or in response to some change. For example, in studies where an intervention or perturbation may exist, like inducing a phase shift with jet lag (Vosko et al., 2010; Waterhouse et al., 2007; Eastman and Burgess, 2009), more data is needed after the perturbation to actually quantify the phase shift. Time-frequency methods (the balance of signal information from the time domain and frequency domain) can be used to highlight such changes but face limitations if certain thresholds of sampling rate and sampling length are not met. The time domain is the classic representation of sample amplitudes taken across time, whereas the frequency domain is the representation of the different frequencies that exist in a signal. There is an inherent trade-off in signal representation between the time and frequency domains, otherwise known as the Uncertainty Principle (Folland and Sitaram, 1997). It can be succinctly described as follows: it is not possible to know both the amplitude and frequency of a signal with certainty at any arbitrary moment. Essentially, instantaneous signal in the time domain gives no information about frequency composition, and instantaneous signal in the frequency domain gives no information about amplitude of the signal at specific times. Time-frequency methods such as the short-time Fourier transform (STFT) (Gnyubkin, 2010; Xu et al., 2020), wavelet transform (WT) (Price et al., 2008; Leise, 2015; Nounou et al., 2012), and Hilbert-Huang Transform (HHT) (Huang et al., 1998) are capable of highlighting the analyses where one is interested in how frequency properties of a signal change through time. However, a better time resolution necessitates a poorer frequency resolution and vice versa. While the time-frequency methods can allow researchers to hone in on the cyclic characteristics of a signal pre/during/post a perturbation, a greater sample length is needed to achieve statistical power depending on which parameters/variables are being compared. For example, if a hypothesis were that circadian temperature amplitude/stability decreases after traveling across time zones (i.e., jet lag), then *at least* 2 days of temperature data would need to be sampled before and after traveling as a minimum threshold for assessing changes in frequency. This is not to imply that stability always re-emerges within 2 days (which would only happen after very small shifts), but rather is meant to highlight a spectral “floor” of data needed to infer cyclic parameter changes. The intuition for this goes back to the Uncertainty Principle: because characteristics of a signal can vary in both frequency composition and time (especially in signals with additional noise), sampling for only a day after a perturbation makes it difficult to disambiguate between a cycle and a linear trend in signal amplitude over the duration of the day. Two days would provide a minimum assurance that the 1-day cycle has persisted for 2 days. However, similar to the Nyquist Rate, this does not guarantee that the signal of interest can be extracted from noisy data, so repeated measures across additional days provide greater precision when measuring oscillation parameters, as in period and phase.

#### Data Orthogonality

Having multiple data streams can equip a researcher with the ability to better account for variance/behavior of the observed system (Bae and Androulakis, 2018; Díez-Noguera, 2013), just as one might measure expression of both *Per* and *Bmal* genes to establish the phase of a given circadian oscillator. Sometimes, measuring multiple outputs (data modalities) like this provides confirmation of an expected pattern, as with the *Per* and *Bmal* example. Often though, modalities are chosen for their lack of association. The more independent information a new modality provides compared to those already included in a dataset, the additional uniqueness that modality then adds to contextualizing a system. When one seeks to reduce the amount of data being processed, then the data streams/dimensions should be chosen so that they do not share a large amount of linear variance with each other. When there is zero linear variance shared, the two modalities are said to be orthogonal, as in being contained entirely on uncorrelated axes. Many methods exist to establish orthogonality of modalities. *A priori*, the data streams can largely be decided based on prior work, domain expertise, and feasibility of acquisition. *A posteriori*, methods such as principal components analysis (Jolliffe and Cadima, 2016) or Graphical LASSO (Friedman et al., 2008) can be used to evaluate a reduced set of linearly independent features. (Note: Graphical LASSO is typically used when the amount of variables is larger than the number of samples, such as in the case of tissue gene expression data.) It is important to note that these methods will only identify potential linear relationships. Any nonlinear function between 2 variables x and y that is actually causal, for example $y=x^{2}$ , would be identified as likely being linearly independent from each other despite the fact that there is a direct mapping between them. As such, researchers should practice caution when making assumptions of linearity with their variables of interest. In addition, sometimes it is useful to keep variables that are not wholly independent because there are specific instances in which their independence changes and that is the issue of interest. For example, when studying jet lag, many circadian outputs should be correlated before the circadian shift. If one wants to study how internal desynchrony emerges and then resolves following a shift, then an experimenter would not want to throw out variables with some correlation within the dataset. Their domain expertise informs them that that correlation is itself a feature of interest that will change in time and generate its own meaningful signal to be analyzed.

### Imputation

#### Sources of Missingness

As data grow in observations and time resolution, the likelihood of missing data increases. For example, researchers may occasionally fail to manually record observations at finer time resolutions. Devices, while largely immune to human error, may fail to record data for many reasons, some of which include limitations in storage, depleted battery life, or issues with wireless connectivity. Unbeknownst to the researchers, devices may further engage in “under the hood” arbitration of data quality, where data deemed by the device as implausible or too noisy will be marked “missing.” One may manage these missing observations such that they can retain as much data (and by extension, statistical power) as possible through *data imputation*. Data imputation involves replacing missing or unknown observations in a dataset with plausible values (Donders et al., 2006).

**Figure 2.**
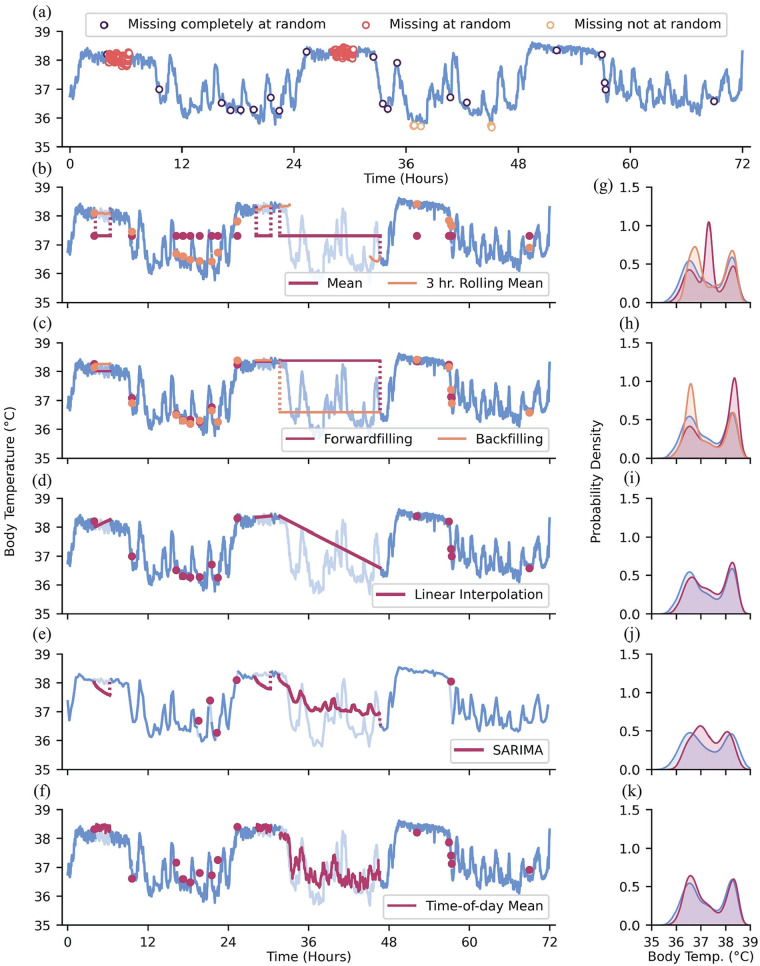
Types of data missingness and methods of addressing missingness. (a) Data MCAR, MAR, and MNAR depicted as open scatter points. (b) Mean and rolling mean imputation; pale blue lines: artificially missing observations. (c) Forwardfilling and backfilling imputation. (d) Linear interpolation imputation. (e) SARIMA imputation with data downsampled to 288 observations for computational efficiency. (f) Time-of-day mean imputation. (g-k) Distributions of ground-truth data values (blue) and body temperature values with imputed values obtained from the methods on the left-hand side of each corresponding subpanel.

There are different qualities of data missingness: data may be “missing completely at random” (MCAR), “missing at random” (MAR), and/or “missing not at random” (MNAR) (Donders et al., 2006; Moritz et al., 2015; Newgard and Lewis, 2015; Papageorgiou et al., 2018). We compare these cases using the example of wearable devices recording actigraphy data. For MCAR data, there is no systematic reason for missing values, and missing values are independent of other variables. An example of data MCAR is intermittent, seemingly random failures of wearable sensors to record actigraphy data. When data is MAR, missingness is correlated with other variables. In univariate time-series data, time is the only other observable variable that may influence missingness (Moritz et al., 2015). For example, individuals may be more likely to utilize wearable devices on non-work days when they are not restricted by workplace dress or safety codes. The pattern of data missingness becomes evident in this scenario since it stratifies data by day of week or weekends and weekdays. In contrast, data that are MNAR are correlated with unknown or unobservable factors, such as in the event where we cannot observe rule-based, “under the hood” data elimination. For example, large instantaneous changes in actigraphy may be flagged by the sensor as implausible anomalies and thus all values above a certain threshold are systematically discarded. Unaccounted for sociodemographic factors may also play a role in MNAR data, which is worth keeping in mind when thinking about the generalizability of findings from data mining efforts. For example, it may be infeasible for individuals with little disposable income to utilize wearable devices compared to those with greater access to disposable income. The non-random structure of missingness may not be captured within the dataset being analyzed (if, to continue the disposable income example, socioeconomic status is not curated within the dataset), but may be able to be assessed anyway if the metadata of the dataset are available (e.g., if the site of collection is an affluent hospital system, socioeconomic imbalance may be inferred, if not recalculated by population distribution statistics). We recapitulate these modes of missingness in Figure 2a.

#### Single Imputation

Time-series analysis methods often require continuous, complete datasets. Because missing or unrecorded data are not uncommon in longitudinal data, analysis of only “complete” data may lead to great data attrition or biased statistical analyses if data are missing in a systematic manner (Graham, 2009; Zhang, 2016). Data imputation offers methods for addressing these missing values and retaining a more complete dataset. The simplest approach to imputation may be single imputation, which involves replacing missing values with a single value. A common method for single imputation involves replacing missing values with a measure of central tendency (e.g., mean or median) from the non-missing values (Graham, 2009; Zhang, 2016; Dziura et al., 2013; Figure 2b and 2g). Population or sample measures of central tendency may be used, though imputation using sample-specific values (i.e., imputing missing observations from an individual using their own non-missing data) is often superior to population values (Engels and Diehr, 2003). The observation prior to a missing value(s) may be carried forward to fill in missing values, which leverages the dependent nature of time-series data; back-filling values may be warranted if missing values appear at the start of a time-series (Moritz et al., 2015; Dziura et al., 2013; Figure 2c and 2h). It is important to note that imputation approaches that duplicate the same values many times can bias and artificially remove variance of the dataset, especially when a large number of data points are missing (i.e., with enough filling in by the mean, one is eventually meaningfully reducing the standard deviation of the resultant filled dataset; Haziza, 2009). Moreover, if the time-series has meaningful oscillations (e.g., daily rhythms), then imputing with the mean may corrupt the pattern even when it does not meaningfully change the gross statistical description of the dataset.

#### Time-Series-Friendly Imputation

Slightly more complex methods can help maintain the variability and trends in time-series data: linear interpolation can be used to estimate values over short stretches of missingness by drawing a straight line between the non-missing points immediately preceding and succeeding the stretch of missing values; however, this approach may not be optimal for longer stretches of missing data, especially in a context where the missing measurements can be expected to be changing nonlinearly or non-randomly through time (Weed et al., 2022; Kornelsen and Coulibaly, 2014; Junninen et al., 2004; Figure 2d and 2i). To impute longer stretches of oscillating data, one may replace missing data with data from the same time periods from the previous day or with aggregate (e.g., mean, median) time-of-day matched values from multiple days (or whatever the periodicity of the data may be; for example, if someone is missing minute-level data all day on a Tuesday, it might make sense to impute that missing day by inference from that participant’s previous Tuesdays’ values at those missing times, rather than global averages or even inferences from all days of the week) (Tonon et al., 2022; Weed et al., 2022; Figure 2f and 2k).

Other approaches that take into account the local, potentially nonlinear structure of time-series data include imputation based on moving averages (Wijesekara and Liyanage, 2020) and Seasonal Autoregressive Integrated Moving Average (SARIMA) models (Duangchaemkarn et al., 2022). Moving averages use surrounding, non-missing observations to impute a central missing value. This method can be applied across a time-series to impute shorter gaps in the data (Figure 2b and 2g). However, unless one utilizes previously imputed values, there may not be any available data to impute with across long stretches of missingness. SARIMA models, more commonly discussed as statistical analysis tools (refer to subsection Statistical Models for more in-depth description of a SARIMA model), can be used to predict missing values based on past, representative values (Figure 2e and 2j). SARIMA models may infer which values are more representative and likely at a given point by utilizing a user-specified “seasonality” component, which is an encoding of the expected periodicity of the data.

#### Multiple Imputation

The inferences drawn from datasets filled in using single imputation may heavily rely upon the imputation method used. If that method turns out to generate artifacts that affect analyses, then these methods may lead to false precision of results (Donders et al., 2006; Li et al., 2015). To reduce the potential harm of any one imputation choice, multiple imputation is used to compare the outcomes from several imputations of the same dataset and their subsequent (identical) analysis (Donders et al., 2006; Li et al., 2015; White et al., 2011). From these analyses, the parameters (e.g., the MESOR of a biological time-series) obtained from each imputed dataset can be aggregated into an estimate that is more likely to contain the true value of the parameter than any one imputation method alone. Some programs (e.g., SAS) will have multiple imputation functions built in. Analyses are run on each imputed dataset (one set per imputation method used) and then the estimated outcomes can be presented as a distribution based on the methods used. The intention is to accurately reflect uncertainty and to ensure the results of the analysis are not overly shaped by the choice of imputation method.

For multivariate datasets, more informed (albeit more complex) imputations can be made by considering the values of the adjacent non-missing variables and what value the missing value tends to take on when those specific non-missing values are present. In other words, imputed values of multivariate datasets can be obtained by conditioning the imputation on the distribution of other variables in the dataset: if the value for modality *A* and *C* are known where the value for modality *B* is missing, then various methods can be used to make a probabilistic inference of the missing value in modality *B* based on other places in the data where all three modalities are present (e.g., if heart rate is higher than usual at a time when activity is missing, one may infer that activity is likewise probably higher than usual). For univariate data, the approaches to imputation avoid this complexity since time is the only other observable longitudinal variable. Even so, there remain more and less complex methods of imputing multiple plausible datasets for multiple imputation. We argue that even the simpler method of imputing values, such as using time-of-day means or medians, can make for a valuable first-pass at generating multiple imputed datasets for multiple imputation. In the interest of space, we do not explore more complex imputation methods here, but see Adhikari et al. (2022) or Kazijevs and Samad (2023) for further information.

### *p*-Values and Effect Size

For biologists who work with vast amounts of data, 2 issues lead to challenging the way p -values are intuited. First, with larger N s per comparison, statistical tests are more likely to reveal significance due to the commensurate increase in statistical power alone. Second, having more data often means one can make more different comparisons; with a false discovery rate of 5% ( p = 0.05), we expect many false “significances” when making hundreds or thousands of comparisons. This is a problem that was faced by genetic researchers years ago (as in testing the significance of thousands of positions in the genome between 2 phenotypes), but which is becoming more common as large datasets become available in more biological domains. Having longer time-series that are sampled frequently or more individuals in a study can make it much easier to reach significance in any comparison. Take, for example, CBT data sampled from a mouse every minute. The true mean of this mouse’s CBT distribution is 36 °C. If a second mouse has a true mean CBT of 36.05 °C, as more data are sampled from each mouse, at some point a statistical test will reach significance ( p< 0.05) for testing a difference of means. Even if we correct for multiple comparisons appropriately, we will simply find more significant effects with more data. The difference between the true means for the 2 mice might be highly significant, but is quite small and arguably not of practical relevance. The (corrected) p -value only captures how confident we are in rejecting the null hypothesis, but it does not reveal the practical utility of that rejection. One solution to both problems is therefore to compare not just p -values, but the combination of p -values and effect sizes.

Effect sizes are used as approximations of the “usefulness” of a finding. That is, they provide estimations of how far apart 2 distributions are from each other. A p -value reveals a significant effect, but an effect size reveals the *magnitude* of that effect. If we refer back to the example of the 2 true CBT means of the mice, the effect size (roughly, the difference of the true means) has not changed, but we have sampled enough data (and amassed enough statistical power) to identify a difference. Because significance can often be found in large datasets due to statistical overpowering, we highly recommend adopting volcano plot-related practices from genomics and reporting effect sizes along with significance and test statistics so that reviewers and/or readers may infer how impactful a finding really is (Wodrich et al., 2021). There are many effect size estimates available; for the sake of brevity, we will list only Cohen’s *d* (Cohen, 1988), odds ratio (Szumilas, 2010), and Cliff’s δ (Cliff, 1993) and encourage interested readers to investigate the assumptions underlying their chosen effect size measure(s).

### Noise

We can imagine the observation of a time-series as sampling from an underlying process that generates that time-series. In the evolution of temporal data, there are components of the process that change in more expected ways (e.g., the circadian oscillation of CBT), components that are deterministic but unobservable that affect the signal (e.g., transient perturbations in core temperature due to changes in physical activity), and components that are due just to a researcher using an imperfect sensor to detect a signal.

The processes observed from experimental time-series data can be interpreted in 2 primary ways: (1) deterministic (Birkhoff, 1927) or (2) stochastic (Doob, 1990). A deterministic process is a process in which there is no randomness involved (i.e., if the underlying equation of the process is known, it is possible to predict all values of a process; for example, a pure sine wave). A stochastic process is a process in which there is at least one random variable that affects observations. The random variable need not reflect the “true” behavior of the value of interest in a study, but could represent any unknown random quantity that affects the value of interest. Two pertinent examples of random variables in time-series are measurement noise and process noise (Kalman, 1960). Measurement noise is uncertainty in the measurement of the observed variable due to variability in precision, such as a test’s confidence in the concentration of cortisol is ±2 µg/dL and a sensor can estimate temperature within ± 0.25 °C. Process noise is uncertainty in the state variable. The state variable is something that is hidden from the observer (e.g., blood cortisol concentration when only saliva is measured, sleep state when only actigraphy is available). Any variable, both exogenous and endogenous, that cannot be accounted for but can impact variable of interest can be considered process noise (e.g., mild acute stress responses might not be measured across a day, but would nevertheless lead to transient increases in cortisol, and so affect the measured shape of that day’s cortisol concentrations). It is of note that process noise is not necessarily “useless” perturbation that affects the variable of interest. Many biological conditions and processes have varying magnitudes of process noise and, when all else is accounted for, these magnitudes may differentiate levels of system complexity (Costa et al., 2002; Mazzocchi, 2008). In practice, nearly all time-series contain stochasticity due to uncertainty in the estimation of an underlying “true” value. It is then valuable to identify if one’s noise removal process is accidentally omitting process noise (which may assist in understanding system complexity) along with random noise. While parameterizing process noise can be difficult, there are many methods available to handle random noise. We provide some examples in the following sections.

#### Noise Removal—Time Domain

**Figure 3.**
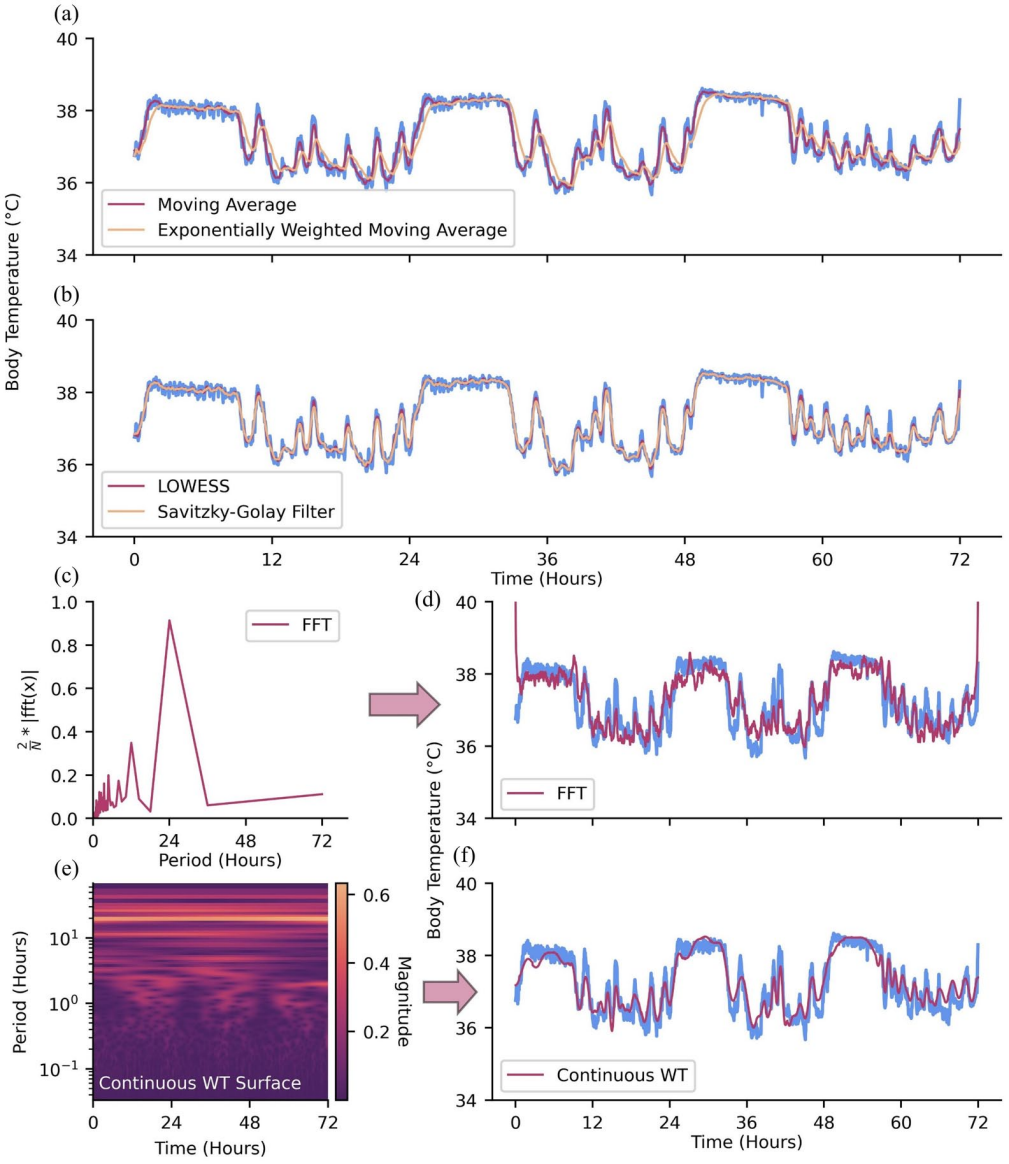
Methods for de-noising data. (a) Moving average and exponentially weighted moving average. (b) LOWESS and Savitzky-Golay filter. (c) Continuous WT and (d) continuous WT coefficient magnitudes prior to zeroing coefficients of amplitude <0.20. (e) FFT and (f) FFT scaled amplitude prior to zeroing coefficients of amplitude <0.40.

After filling in missing values, one may remove noise from the dataset. One straightforward approach to removing instantaneous, high-amplitude noise (i.e., spiky, high-frequency noise) relative to the rest of the time-series and highlighting long-term fluctuations is with a moving average filter, where data is averaged over a moving window of pre-specified size (Alsberg et al., 1997; Nounou et al., 2012; Wang et al., 2016; An and Stylios, 2020; Figure 3a). This is a simple example of a low-pass filter, which serves to attenuate short-term, high-frequency fluctuations in data. One may also use this moving window approach but calculate a moving median to further attenuate the effect of extreme outliers (Arce, 1998). Other moving average methods may weigh observations unevenly, often giving more influence to recent observations or observations closer to the center of the moving window (e.g., how you slept 10 days ago is informative for how you feel today, but less so than how you slept last night) (Hyndman et al., 2008; Nounou et al., 2012; Ghalyan et al., 2018; Figure 3a). While moving-average methods serve to attenuate high-frequency noise, there exist filters to attenuate low frequencies (i.e., high-pass filters) and frequencies within certain ranges (i.e., band-pass filters); one’s de-noising needs depend on the problem at hand (Xie et al., 2021).

Another approach to de-noising data is by fitting neighboring observations to regression models (Cleveland and Loader, 1996). Here, subsets of neighboring observations within a window of pre-specified size are fit to a regression with the goal of obtaining a de-noised estimate of the center observation. Compared with moving average methods, regression models for de-noising tend to maintain local fluctuations in the time-series (contingent on regression order) rather than long-term trends. Two examples of local regression methods are LOcally Weighted Scatterplot Smoothing (LOWESS), which is a weighted linear regression, and LOcally Estimated Scatterplot Smoothing (LOESS), which is similar to LOWESS, though the former typically fits observations to a second-order regression (Moosavi et al., 2018; Figure 3b). A more general instance of regressions for de-noising is the Savitzky-Golay filter, which fits neighboring observations to an unweighted, nth-order polynomial (Savitzky and Golay, 1964; Komsta, 2009; Azami et al., 2012; Figure 3b). A Savitsky-Golay filter of order zero is equivalent to a simple moving average, as a constant value is fit to the observations in a given window. The extent to which the variability of data is maintained by these localized regression methods can be manipulated by specifying the order of the regression and the window size in which the regression will occur.

#### Noise Removal—Frequency Domain

In biological rhythms research, it is usually the case that time-series of interest are comprised of oscillations that occur at certain frequencies (or periodicities). Some frequency components of these data may be more prominent than others, such as the 24-h circadian rhythm characteristic of many biological processes. When one knows which frequencies are of interest and which are likely to be noise (or at least, not of interest), the contributions of unwanted frequencies can be addressed and attenuated via the frequency domain rather than the time domain.

Signal processing methods used for time-frequency analysis, such as the Fast Fourier transform (FFT; Walker, 1997; O’Haver, 1997; Wahab et al., 2021) and WT (Ergen, 2012; Taswell, 2000), can also serve as frequency domain-based de-noising tools (refer to subsection Fast Fourier Transform and Wavelet Transform for time-frequency methods as tools for analysis). In the case of the FFT, the time-series is first converted into the frequency domain (Figure 3c). Undesirable frequency components can then be attenuated before inverting the FFT to recover the de-noised time-series (Figure 3d). The WT (continuous or discrete) serves a similar role, with a notable difference being that it converts the time-series into a time-frequency representation (Figure 3e). Low-magnitude wavelet coefficients can then be attenuated before inverting the WT to recover the de-noised time-series (Figure 3f).

### Outliers

Outliers are the clearest example of those topics mentioned in the Introduction that are not at all specific to Big Data—everyone reading this has dealt with outliers. However, it bears special consideration, as any cleaning done in Big Data must be algorithmic—it cannot be done by hand due to the scale of the data being cleaned. Even seeing enough outliers to understand what should be cleaned can be hard, as seeing all of any Big Data object is its own challenge. Therefore, we expand on systematic ways of considering outliers here. The concept of an “outlier” has been succinctly described by Hawkins (1980) as an observation that deviates so greatly from other observations that it appears to have been generated by a different underlying mechanism and is not representative of the process being measured. Outliers are, in a way, closely related to noise. Noise can be characterized as all the underlying processes that influence observations that would best be attenuated to amplify the underlying signal of interest (Ranga Suri et al., 2019). While both outliers and noisy observations contribute to deviations from the researcher’s idealized dataset, outliers tend to deviate far more from other observations than noisy observations. Detection and subsequent interrogation of outliers may reveal important (and often interesting) information about the data, such as incorrect distributional assumptions or distinct subcategories of observations (Domański, 2020; Smiti, 2020; Kotu and Deshpande, 2014).

**Figure 4.**
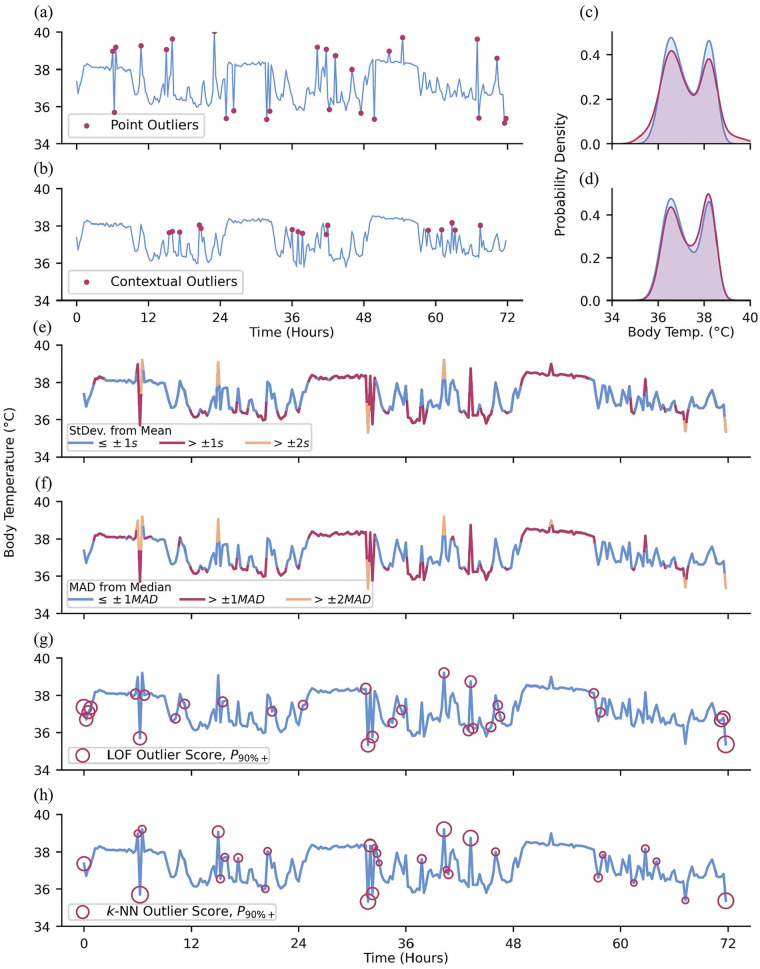
Types of outliers and methods for outlier detection. (a) Red dots: artificially added point outliers and (b) local outliers; the original minute-level data was downsampled to 288 observations to emphasize the contribution of outliers to the (c and d) distribution of observations. (e) Downsampled data colored by standard deviations of difference from the sample mean. (f) Downsampled data colored by MADs of difference from the sample median. (g) Outlier detection using LOF and (h) k-NN, using 5 neighbors and showing the top 90th+ percentile of “outlier-ness.”

It is especially important to detect and deal with outliers prior to analyses, as outlier values may greatly impact statistics computed from the data, such as the commonly reported sample mean (Huber, 1981). There are 3 commonly cited types of outliers: point, contextual, and collective outliers (Chandola et al., 2009; Braei and Wagner, 2020). Point outliers are observations that are out of range with respect to all other observations in a dataset (Figure 4a and 4c), while contextual outliers are anomalous with respect to the neighboring observations, but might not be out of range for the whole dataset (Figure 4b and 4d). Contextual outliers are especially common in oscillating data, where the local expected range may be substantially smaller than the full dataset range (we detected daytime fevers during the 2020 COVID-19 pandemic by this method: elevated daytime skin temperatures associated with fevers were well within the normal range of all data, but very high and easily detectable compared to the much smaller range of waketime skin temperatures; Smarr et al., 2020). Collective outliers are sequences or clusters of observations that together deviate from the overall dataset; the individual observations that comprise collective outliers are not necessarily themselves point or contextual outliers but collectively deviate from the norm. For example, charging one’s wearable device often acutely raises the temperature of the device. This rise in device temperature may be captured and recorded as a sequence of (erroneous) skin temperature observations that steeply rise before being cut off, as the device ceases to record any further data. In the following section, we cover approaches to managing point and contextual outliers as the identification of collective outliers is a more complex task that may require more domain-specific knowledge.

#### Simple Heuristics

Statistical approaches to outlier detection are a common method of detecting point outliers in data (Kriegel et al., 2010). One common rule-of-thumb approach to detecting point outliers involves discarding observations exceeding ±2 to 3 standard deviations from the sample mean (Grubbs, 1969; Hodge and Austin, 2004; Cousineau and Chartier, 2010; Leys et al., 2013; Figure 4e). While this approach is a compelling heuristic, it relies on having prior knowledge of the underlying distribution of the data (Domański, 2020; Alimohammadi and Nancy Chen, 2022). Furthermore, this approach incorporates all data, including outliers, into the sample mean and standard deviation. As a result, the bounds for non-outlier data may be too permissive of extreme values due to their influence on the mean and standard deviation which comprise those bounds.

Rather than sample means and standard deviations, a more robust approach is to use the median and mean absolute deviation (MAD), respectively (Leys et al., 2013; Figure 4f), removing or proportionally penalizing observations that deviate more than an arbitrarily predetermined threshold (e.g., ± 3 standard deviations or MADs; Mehrang et al., 2015). Similarly, one may utilize the interquartile range (IQR), where values IQR*1.5 less than quartile 1 or IQR*1.5 greater than quartile 3 are deemed outliers (Smiti, 2020; Hodge and Austin, 2004).

#### Outliers With Respect to Time

Local observations that deviate from neighboring points may not be extreme enough to be detected by the above methods (Blázquez-García et al., 2021). In such cases, these methods can be used within moving windows to allow for local resolution. Better still are approaches that rely on the localized structure of a time-series to identify contextual outliers (Kotu and Deshpande, 2014). Local outlier factor (LOF; Breunig et al., 2000; Chen et al., 2010) quantifies the “outlier-ness” of an observation as a function of how isolated it is compared with its *k*-nearest neighbors (Figure 4g). LOF is an example of a density-based approach to outlier detection. On the other hand, *k*-Nearest Neighbors (Ramaswamy et al., 2000; Hautamaki et al., 2004; Yu et al., 2014; Dang et al., 2015) is a distance-based measure, where a greater average distance between the observation and its *k*-nearest neighbors suggests greater outlier-ness (Figure 4h). Time-series data in particular may benefit from methods to identify contextual outliers since the expected range of observation values may be highly dependent on the time of data collection.

#### Expert Input

Finally, domain expertise may be used for rule-based outlier detection to filter out improbable values that may have occurred due to instrumentation or researcher error (Sejr and Schneider-Kamp, 2021; Salgado et al., 2016). For example, sub-physiological values of skin temperature or heart rate as recorded by a wearable device may indicate that an individual recently removed the wearable device as the device continued to record. Recognizing these implausible values would require the input of individuals who have sufficient knowledge of the system being measured to make an appropriate inference (in this example, human physiology).

#### Beyond Outlier Removal

Once outliers have been identified and removed, one must decide whether or not to impute any newly missing values. Important factors to consider when making this decision may include data attrition in instances where complete data is required and the quality of the data imputations would be based upon. Subsection Imputation details possible methods of imputation.

### Phase Alignment

To compare multiple time-series, one may require more information than just timestamps. While data may be aligned in “clock time,” this does not guarantee phase alignment. For example, long-distance travel is known to induce physiological jet lag, where internal biological rhythms are out of phase with the external environment and with each other. Two individuals subject to identical external environments but with different chronotypes may be consistently out of phase with each other with respect to physiology even without jet lag (Baehr et al., 2000; Lack et al., 2009). The more misaligned individuals that are combined into an average, the less representative is that average, with the extreme example being that the mean of many misaligned sine waves is a flat line. This suggests that accounting for phase differences is necessary to allow for the comparison of other characteristics of time-series, such as differences in amplitude at a particular phase, especially in large datasets where a wide range of phase alignments are likely to exist.

While the degree to which a time-series is periodic may depend on one’s specific method for determining periodicity, intuitively a periodic time-series can be expected to repeat at regular intervals (Deckard et al., 2013). This property of periodic time-series allows them to be effectively aligned with other time-series of similar periodicity. For example, a simple, first-pass approach to alignment of periodic time-series includes cross-correlation. The cross-correlation of time-series allows one to find an optimal time lag that maximizes the correlation between the 2 signals (Dean and Dunsmuir, 2016; Menke, 2022). This time lag can then be applied as an offset to one of the time-series to align them at the point of maximal cross-correlation, such that they are more closely aligned with respect to phase. For less stably periodic (or even aperiodic) data, dynamic time warping (DTW) can provide an alignment between 2 time-series such that one time-series can be compressed or dilated to best align (minimize difference or error) with the other (Müller, 2007; Giorgino, 2009; Skutkova et al., 2013; Smarr and Kriegsfeld, 2022). Furthermore, the DTW provides a distance value between time-series, which can be used as a similarity metric. Time-series that share common defining features (such as the characteristic troughs and peak of a QRS complex of a sinus rhythm) may be aligned using functional data analysis approaches (Wu et al., 2024). In these cases, one may prioritize alignment based on these defining “landmarks” that are present across all observations. In cases where prominent landmarks may not be present and there is little phase shift between observations such that the mean time-series across all observations is fairly representative of individual observations, alignment by minimizing the distance between each time-series and the mean time-series across all observations may be a feasible, intuitive approach to alignment.

### Data Analysis Techniques for Time-Series

Once the proper preprocessing due diligence has been performed on the time-series of interest, it is then possible to transition into utilizing analysis techniques that can extract meaningful representations of cyclic data for hypothesis testing. The following methods, while certainly not exhaustive, are aimed at collecting a set of variables that can be used for statistical comparisons between an experimental time-series and a possible control time-series.

#### Cosinor Regression

Cosinor regression is a powerful, interpretable tool to parameterize an oscillatory signal into periodicity, acrophase, amplitude, and midline estimating statistic of rhythm (MESOR) (Cornelissen, 2014; Bingham et al., 1982). Cosinor methods also provide confidence intervals on the estimated parameters of the model, allowing for relatively straightforward ways of testing hypotheses related to signal rhythmicity. A major assumption of cosinor methods for realizations with multiple cycles is that the parameters of the function do not vary in time (e.g., the signal is assumed to be stationary; that is, has constant values for the parameters), and that the signal has a monotonically increasing, linear evolution of phase. While there are nonlinear extensions of this method (Marquardt, 1963), they are less easily understood. The ease of the cosinor method is the simple interpretation of its parameters. This method is often used when estimates of periodicity and/or phase are known. For example, cosinor-derived circadian amplitude of melatonin and cortisol was found to be lower in heart failure patients compared to control (Crnko et al., 2023).

#### Fast Fourier Transform and Wavelet Transform

While cosinor methods are typically used to test for hypothesized periodicities (e.g., circadian/ultradian rhythms), FFT and WT can decompose a signal into (theoretically) all underlying periodicities. FFT and WT also make assumptions of linear evolution of phase. However, FFT removes all time information and in return provides frequency information about a signal (Walker, 1997; Gnyubkin, 2010). This can be an optimal tool for individuals who hypothesize that there may be hidden periodicities in longitudinal data (e.g., multiple ultradian rhythms in a novel dataset that are not necessarily multiples of a dominant cosinor rhythm). However, since the FFT is unable to disentangle rhythms that appear/disappear depending on a larger rhythm (e.g., ultradian rhythms that only appear during a certain phase/window of a circadian rhythm), it is not necessarily able to capture a time-dependent hidden periodicity. The WT accounts for time-dependent rhythmicity and can show how frequency content changes with time, but the interpretation becomes more difficult (Walker, 1997; Liò, 2003; Leise, 2013).

**Figure 5.**
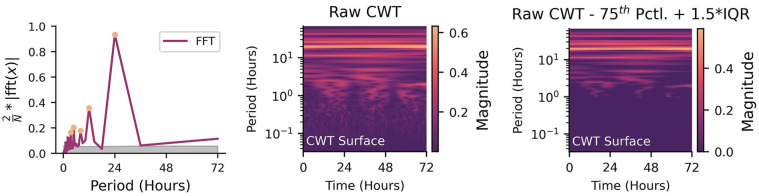
Left: Identifying significant peaks from a periodogram (orange circles) when compared to the null hypothesis noise floor of multiple shuffles (gray shaded area). Middle: Unfiltered logarithm of the absolute amplitude of the continuous wavelet transform for three days of data. Right: Same continuous wavelet transform, but differenced against 1.5 times the interquartile range above the 75th percentile. Non-zero values indicate amplitudes that exceed the threshold for significance.

In the 2D case (FFT), the output is power at different frequencies. In the case of a signal with a strong 24-h periodicity, there would be a peak at that periodicity in the FFT. If one wanted to test the null hypothesis that there is no 24-h rhythm, they would compare the power at the periodicity of interest to the power of the FFT at relatively flat regions (noise) by either doing a simple test to see if the 24-h power is about 2 standard deviations outside of the noise (95% confidence), or greater than 1.5 times the IQR above the 75^th^ percentile, or by randomly sampling from the FFT multiple times to get a null distribution of power to compare against (e.g., Figure 5, Left). The same approach can be used in the 3D case (i.e., frequency × time × power WT surfaces) where the peaks are now “hills.” Comparing the power of the hills against the relatively low power of the flat parts of the WT provides a similar test to the FFT case. Peak finding within specific frequency bands on the resultant surfaces can also be used to identify peaks and troughs of cycles within frequencies, as in detecting daily modulation of ultradian rhythms in mouse locomotion, heart rate, and temperature (Smarr et al., 2019) (e.g., Figure 5, Middle, Right).

#### Mode Decomposition

**Figure 6.**
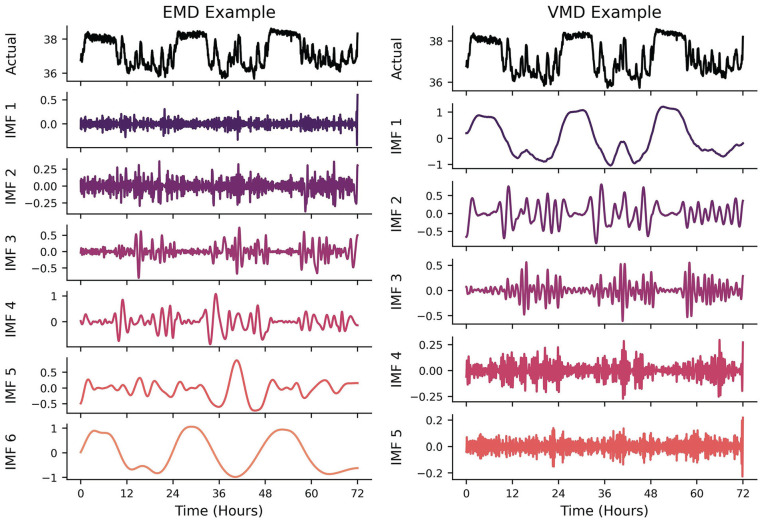
Left: Example of EMD algorithm output. EMD produces IMFs in increasing frequencies. As it removes the higher-frequency peak/trough envelopes detected in the time-domain, the IMFs capture lower- frequency oscillatory components. Right: VMD algorithm output. While VMD can concurrently generate the different underlying IMFs of a signal, most algorithms will output the lowest frequency IMF first.

Mode decomposition (MD) attempts to decompose a time-series into a subset of intrinsic mode functions (IMFs) that, when summed together, reconstitute the original signal within some margin of error. As the FFT and WT can be thought of as a summation of multiple underlying rhythms in the frequency domain, MD can be thought of as rhythm summation in the time domain. The different versions of MD (Empirical, Variational, etc.) were all designed to overcome the issues of the FFT and WT in the presence of signals with nonstationarity and nonlinearity. This method is highly recommended for researchers who would want to disentangle different rhythms in the time domain rather than having to create a spectral filter in the frequency domain. It is incredibly powerful if underlying periodicities are already known *a priori* and the purpose of using the MD algorithms is to *highlight* the nonstationarity/nonlinearity of the underlying signals (e.g., separating a circadian-dependent ultradian signal in the time domain and quantifying how its phase evolution is not linear in time). Empirical MD (EMD) is a data-driven approach: there are no theoretical harmonic/oscillatory components that are being fitted to the signal of interest (Quinn et al., 2021). It makes no assumptions about the underlying generator of the signal, making it relatively easy to use (at the risk of requiring more in-depth interpretations!). This method is typically used in instances where the number of underlying oscillations is not known *a priori* and there is some interest in adaptability to nonstationary signals (Ortiz et al., 2020). The algorithm iteratively calculates temporal envelopes (i.e., outlines the extremes) around the original signal based on peaks and troughs and then differences these envelopes from the original signal. This process is repeated until a locally smooth IMF is constructed prior to calculating the next IMF (e.g., Figure 6, Left). For example, a longitudinal signal may have multiple ultradian rhythms, a circadian rhythm, a weekly rhythm, and a seasonal rhythm (to name a few). EMD would theoretically be able to disentangle each of these rhythms from each other and return each time domain IMF back to the researcher. This is a viable option for researchers that may think a spectral filter (e.g., lowpass, bandpass, highpass) may be inappropriate for their data. One could interpret this algorithm as iteratively filtering out different frequencies of oscillations, making it useful for interrogating only the oscillation of interest (e.g., a roughly 24-h cycle).

Variational MD (VMD) takes a predetermined number of IMFs prior to attempting to find the envelopes of the power spectrum that best capture the time-varying signals (Dragomiretskiy and Zosso, 2013). While EMD operates recursively on the time domain signal, VMD concurrently and adaptively finds the relevant bands of a power spectrum that can reconstitute a signal, thus balancing the errors between them (Figure 6, Right). This method is typically used when the number of underlying oscillations is known or estimated *a priori*, thus constraining the nonstationary/nonlinear analysis to a smaller subset of theory-driven IMFs. This is particularly useful when there are “coupled” rhythmic relationships, such as respiration having an effect on blood pressure waveforms (Hadiyoso et al., 2020)—not only is the blood pressure waveform recovered, but the respiratory waveform can be extracted independently.

A benefit of having decomposed IMFs (possible from multiple variables as well) is that it is then possible to identify potential phase relationships or coupling between different frequencies of signals. If one wanted to find the effect that circadian phase has on the amplitude of an ultradian rhythm, they could compare peak amplitudes in the ultradian IMF with phase extracted from the circadian IMF. The benefit of performing this in the time domain is that potential outliers and nonstationarity can be accounted for, which is more difficult to correct in the frequency domain (Hadiyoso et al., 2020). For example, if the effect of more physical activity on core temperature rising changes as a function of circadian phase, and physical activity is itself primarily following ultradian rhythmicity during wakefulness, the IMFs provide a convenient way of evaluating the ultradian amplitude differences against time of day itself.

#### Networks and Stability

If the primary oscillatory components are identified/known, the topology and/or stability of the oscillations can be interrogated if it is hypothesized that the components are coupled—the change in phase of one signal may impact the phase of another. One such method of parameterizing oscillatory stability is the Kuramoto (1975) model, which supposes that a system of coupled oscillators may have some level of phase dependence. We recommend this model if researchers are interested in *multiple interdependent oscillators*. More specifically, it is capable of modeling multiple coupled oscillators within a system. Two examples are (1) multiple interacting ultradian/circadian hormones within an organism, or (2) many organisms each with their own circadian behavior/phases that interact with each other. Global metrics of stability (Kuramoto Order Parameter) as well as local metrics of coupling (elements in the adjacency matrix) can be used to understand the behavior of a single system (or compare across multiple systems). Such approaches have been used to simulate and interrogate the generation and disruption of suprachiasmatic nucleus oscillations at the micro (Gu et al., 2016) and macro (Goltsev et al., 2022) scales.

#### Signal Complexity and Nonlinearity

**Figure 7.**
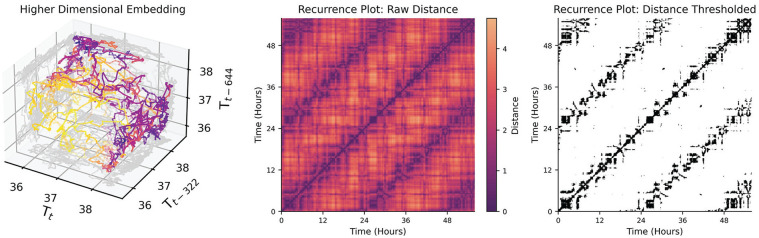
Left: A time-delay embedding of the mouse temperature with lags of 322 minutes between dimensions. A lag of 322 minutes was chosen by the false nearest neighbors algorithm described in Rhodes and Morari (1997). While only three dimensions spatial dimensions can be plotted, the 4th dimension is the color of the points. Middle: A distance plot of the 4-dimensional temperature data where each cell is colored by the distance every point in the 4-D space is to every other point. Lighter regions indicate that the states are further apart; darker indicates that they are closer together. Diagonal lines off of the identity line indicate longer stretches of time when the signal recurs with a similar trajectory to a trajectory in the past.Right: A binary version of the distance matrix where values are threshholded by a distance of 1. Black cells indicate that states are similar enough to be determined a recurrence. This is the data structure commonly used to extract global features of non-linear recurrence.

Rhythmicity can often be interpreted as process functions whose values oscillate in time (e.g., sine, cosine, square, sawtooth). However, states (values) of a system can recur with any arbitrary deterministic and/or stochastic process. For example, while one might expect entrained circadian rhythms to have a periodicity of 24 h, free running rhythms might only have an average period, with some variance day to day, and even bouts of sudden change in period (Mills et al., 1974). Even less regular, ultradian rhythms, as in pulses of cortisol in humans which occur every few hours in the morning (Spiga et al., 2014), may not be truly periodic in many cases, but emergent from feedback systems that are not tightly regulated in time, or may be sensitive to perturbations from other inputs. Recurrence plots are suitable in these instances where metrics of recurrence are of interest but these process functions are not known. A recurrence plot attempts to highlight structure by identifying time-series samples that are close together based on a higher-dimensional embedding of the data. To elaborate on the meaning of structure in a “higher” dimensional time-series: any arbitrary value can recur in a time-series, but historical values can contextualize states (e.g., the state with coordinates of (3, 2) is fundamentally different than the state with coordinates (10, 2) even though 2 is the second value in both coordinates). Each historical (and current) value becomes its own dimension, thus “wrapping” the time-series into a higher-dimensional spatial representation (e.g., Figure 7, Left). The recurrence plot can then be used to visualize the points in time where specific high-dimensional states of the time-series recur with each other. The concept of state then refers to the values of the dimensions after performing a time-delay embedding of the time-series of interest (see Taken’s Theorem (Takens, 1981)). Two components are required to perform a time-delay embedding: a time-delay ( τ ) and an embedding dimension (often denoted as m or p ).

Given a univariate time-series, one can embed the time-series in a higher-dimensional space by using time-lagged values of that same time-series. The time-delay ( τ ) determines how many steps back each embedding dimension will be (Fraser and Swinney, 1986; Sauer et al., 1991; Kim et al., 1999). The number of prior values used determines the embedding dimension (i.e., an embedding dimension of 3 indicates that the state of the time-series in the embedded space can be determined from $\left(x_{t},x_{t-\tau },x_{t-2\tau }\right)$ (Rhodes and Morari, 1997; Cao, 1997; Krakovská et al., 2015). It is then possible to perform recurrence quantification analysis (RQA) on this embedded structure. RQA attempts to featurize aspects of the complexity and/or nonlinearity of a time-series by identifying similar states in the high-dimensional space. Both the raw distance (e.g., Figure 7, Middle) and thresholded distance (e.g., Figure 7, Right) can be used to identify metrics of stability, determinism, and/or laminarity (Webber and Zbilut, 1994; Marwan and Kurths, 2002; Marwan, 2008) that convey unique information about how the high-dimensional signal evolves in time. Since it is capable of encoding information about nonlinear cyclicity, it can be viewed (with a slight stretch of the imagination) as a nonlinear corollary to spectral analysis. Complex hormonal interactions are one such example of nonlinear cyclicity. Insulin and glucagon are produced in response to circulating blood-glucose levels—insulin to sequester glucose into cells and glucagon to release stored glucose (Stagner et al., 1980). Because their concentrations rise and fall throughout the day, spectral analysis would reveal cyclicity. However, the apparent cyclicity is an emergent property of the amplitude relationships between insulin, glucagon, and glucose concentrations. Nonlinear analyses are capable of highlighting rhythmic-appearing phenomena that are actually amplitude relationships arising from complex interactions.

#### Cyclicity in Discrete State Transitions

**Figure 8.**
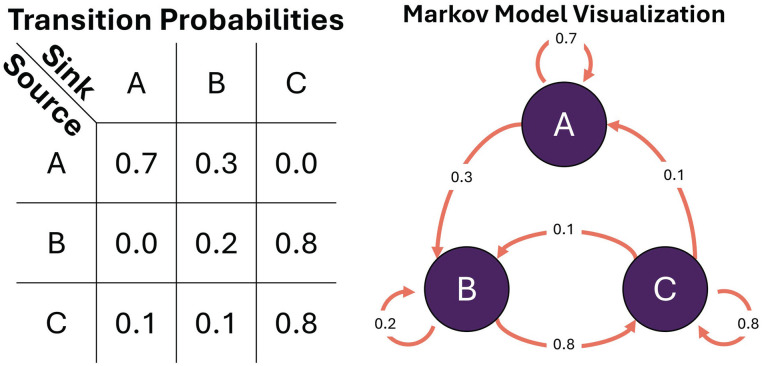
1st Order Markov Model. Left: transition matrix; Right: associated graphical visualization. Nodes/states: purple. Edges/transition probabilities: orange.

Sometimes cyclicity can be modeled as transitions between discrete states, instead of a cyclic evolution of a continuous variable (e.g., having different kinds of sleep patterns across nights; Viswanath et al., 2024). Each state can be imagined as a node in a network, and the transition from one state to another can be represented as a probability. States could be identified by domain-knowledge cutoffs (e.g., actigraphy values above 30 indicate moderate activity states), temporary conditions (e.g., awake vs asleep), or even as the combinations of quantile cutoffs of multivariate data (e.g., 75^th^ percentile of temperature and 25^th^ percentile of activity is unique from 50^th^ percentile of temperature and 25^th^ percentile of activity). The values and directions of these transitions give indications about the propensity for a system to evolve down a path, or to what extent a system is robust to perturbation. The Markov Model is a common tool to evaluate global properties of discrete state transitions (Huang et al., 2018; Perez-Atencio et al., 2018). In a first-order Markov Model, the transition to the next state is dependent only on the current state. That is, there need not be any knowledge about the past except for the current state of a system in order to make a prediction about the next state. This has both positive and negative consequences. On the positive side, it makes the model robust to nonstationarity of variables (Xiao et al., 2005). The intuition behind this is that even if the average of a value changes over time due to an external perturbation (such as physical activity level affecting body temperature), the model can still approximate the likely next transition after the perturbation. However, this only works if the model was able to observe all possible states during the fitting phase. If the new state is physiologically relevant but has not been in observed, the model will just project it to the nearest state. A downside is that unless rhythmicity is explicitly incorporated as a variable into the system (e.g., time of day, light intensity), then global cyclicity (such as that can be modeled with a sine/cosine) cannot be evaluated within the Markov fitting process. This, however, does not preclude analyzing rhythmicity with cosinor, FFT, or WT on the *state* estimates after the model has been fit to the data. A clear benefit when using a Markov Model in instances where a researcher wishes to compare global properties of time-series between samples is that the model essentially transforms an entire time-series into a 2D matrix (Figure 8, Left), where each row and column represent a source node and sink node, respectively (Rabiner, 1989) (e.g., Figure 8, Right). If each node has an important relation to the hypothesis being tested between samples, one could directly compare row-column values (the probability of transitioning from Node A to Node B is higher in the control group vs the experimental group), column sums (Node C is globally more of a sink in the control group), or maximum probability across a row (Node B has a more stable set of transition probabilities in the experimental group) to investigate differences in transition properties. Just as with frequency, time-frequency, and nonlinear transformations, the discrete state transformation allows for novel approaches toward identifying rhythmicity in state changes that may be less obvious in the time domain alone.

### Modeling Temporal and Rhythmic Data

Extracting features from analysis such as statistical modes, rates of change, and/or correlation allows us to use straightforward statistics on time-series-derived features instead of complex statistics on raw temporal data. This extraction of features is called featurization. However, due to the lossy nature of featurization, we often cannot reconstruct the original time-series from features alone. In circumstances where a researcher is interested in time-series reconstruction, forecasting, or causality, the mathematical modeling of the temporal data may be more suitable.

If a time-series itself is a set of observations, the purpose of the mathematical model is to find a likely mathematical construct that describes the evolution of those observations. For observations that are sampled from a pure sine wave, a model can be as simple as the amplitude, frequency, and phase offset of a sine function. Biological systems are more complex than singular, unperturbed oscillations, so we next examine families of models that aim to account for this complexity.

#### Statistical Models

Two fundamental time-series model types are moving average (MA) and autoregressive (AR) (Shumway and Stoffer, 2000; Box et al., 1994). Put simply, while they both use prior observations to predict future observations, the MA model utilizes a linear combination of the historical *error terms* whereas the AR model utilizes a linear combination of the historical observations themselves. These models are useful to researchers if they believe there is both a long-term dependence (such as a rhythm) and underlying time-dependence or noise effects. These models are especially powerful on data that have not been oversampled from a process. For example, if a researcher is interested in some circadian foraging behavior of animals but also in how hourly-level activity measures of animals affect future values, then sampling at the 1-h rate would be practical to create an AR model that incorporates those effects. The most commonly used variant of these models is actually a combination of the two: the Autoregressive Integrated Moving Average (ARIMA) model. The number of autoregressive terms depends both on the apparent complexity of a signal and the frequency of sampling relative to its dynamics. For example, one would have to use many autoregressive coefficients to capture the effect of prior samples from an electrocardiogram (ECG) waveform sampled at 256 Hz because not much information occurs over 1/256 sec. This quality makes ARIMA models less interpretable when the amount of historical terms needed grows, which would be necessary if one wanted to capture rhythmic or cyclic relationships in their data. One would need at least as many lags as the periodicity of their signal. More modern extensions of the ARIMA model have been developed to explicitly capture cyclic components without needing to dramatically increase the amount of parameters needed. While we will not expound on all models that precipitated from MA and AR models, we call out a model that incorporates their linear dependencies with estimates of cyclicity (often termed seasonality in other domains): the SARIMA model (Duangchaemkarn et al., 2022; Perone, 2022). The seasonality component extends the linear model to incorporate historical data from lags that are multiplicatives of seasonalities of interest (e.g., if data is sampled every minute, and it is clear that there is a 1-h oscillation in the data, then the seasonality would incorporate a linear combination of data from time $t_{-60},t_{-2*60},\ldots ,t_{-n*60})$ . The coefficients obtained from these models can be interrogated to determine the lags with the greatest impact on predicting future values.

#### Encoding Phase

Many time-series behave in ways that are dependent on the phase, or state, of exogenous variables (Gander et al., 1986; Rietveld et al., 1993). Depending on the system being analyzed, one could interpret phase effects as values of zeitgebers such as light exposure, ambient temperature, medications, or even day of the week. These variables provide additional context to the behavior of a time-series and can sometimes lead to more robust interpretation of the data. For example, elevated distal skin temperature during the night may appear aberrant compared to prior nights, but knowing that that there may be irregular effects of behavior due to it being a weekend instead of a weekday could provide additional information that the value is not as aberrant as believed, or is at least confounded by external variables. As long as these exogenous variables can be encoded, they can be included in models to potentially improve performance. It is important to note that since they are passed in as variables to the models, they abide by the assumed constraints of the models as well, such as linearity in SARIMA models.

#### Model Validation

Oftentimes, many models are created in order to converge on a proposed optimal solution. It then becomes essential to have pipelines and evaluation metrics that are used to compare the models to each other. The first step is ensure there are a subset of data/samples that the models will never see (often referred to as the “test set”). The selection of this data depends both on how many unique realizations of the model exist (i.e., how many different time-series have been generated from the same system of interest). In the situation where there is only one realization, a test set can be created by taking multiple subsets from the same sample (Sidey-Gibbons and Sidey-Gibbons, 2019). When there are multiple realizations, a researcher can always choose to leave out some last set of data to be tested on from each realization, but could also employ the same resampling technique from the prior method to further extend the test set. For the training of a model, the aim is to minimize the value of a cost function, such as mean squared error, by iterating on different values for model parameters. There are many out-of-the-box cost functions for different purposes (Hyndman et al., 2008; Hodson, 2022), but they all evaluate how well the predicted values from a model actually fit against the observed data. During the testing phase, the predicted values are evaluated against data that the model has never seen. There is a common issue where the error during the training phase of the model is very low, but the testing error is very high. This is an indication of model overfitting. Essentially, the model is capable of fitting the training data so well that it cannot generalize to unseen data. One way to combat overfitting is to reduce the amount of parameters in the model, making it less likely to want to fit to noisier or less informative portions of the data (Ying, 2019). Keeping track of aggregate testing error allows the researcher to then evaluate performance across models and samples in order to hone in on a subset of models that best suit to their needs.

## Conclusion

In this review, we hope to emphasize that the availability of large datasets, analysis tools, and online resources should be empowering—not limiting—for those who are interested in making use of computational methods in the study of biological rhythms. With this in mind, we provided an overview of the diversity of emerging datasets (a small sampling, very far from exhaustive), commonly used data cleaning, alignment, and analysis methods. We emphasize that the material we covered in this review is neither definitive nor exhaustive. Moreover, we intentionally covered well-established, commonly used methods that are available out-of-the-box from commonly used programming languages, or which are straightforward to implement for individuals with limited experience in computational methods. We encourage readers to further explore methods appropriate for their specific use cases.

In addition to these more mechanical insights, we would also like to emphasize that large amounts of data come with pitfalls that are more conceptual. Big Data grant high (often absurd) statistical power, which in turn yield inflated significance in statistical hypothesis testing. This can lead to spurious, false discoveries and wasted time chasing ghosts. It is advisable to use and report measures of effect size to complement statistical hypothesis testing, so that significant findings are accompanied by meaningfully large magnitudes. Related but different, while many individuals may be represented in large datasets, those individuals may not proportionally represent different demographic groups. Nor will every demographic be covered proportionally thoroughly. That is to say, even in Big Data, one should never assume complete coverage of a population, nor misinterpret the presence of significant findings for equal value (e.g., effect size) across diverse populations. For example, some human physiology datasets are generated on-site, leading to the characteristics of the study sample being hyper-localized and findings difficult to replicate at other sites. More broadly stated, the demographic and socioeconomic composition of individuals from one dataset would be challenging to replicate in another. Furthermore, subgroups of individuals may have less available data than others, leading to decreased confidence in the conclusions drawn from analyzing their data. For these reasons, one needs to be cognizant of overstating findings from analyses of large datasets when those findings may not extrapolate to other populations or have lower ecological validity.

Relevant data and powerful methods are necessary, but insufficient, to effectively interpret and understand biologically rhythmic phenomena. It is only when those necessities are utilized by domain experts that phenomenological findings evolve into generalizable knowledge. We hope that broadly introducing datasets and methods to the biological rhythms community will lead to creative problem-solving techniques and novel questions in data that have yet to be interrogated for rhythmic structure. Keeping with our aims, we provide a list of out-of-the-box implementations (in programming languages Python and MATLAB) of the methods outlined in this review (Suppl. Table S1). We also provide a practical flowchart that guides the reader through the process of analyzing time-series data (Suppl. Fig. S1). We again stress that these resources are neither definitive nor exhaustive but simply serve as useful quick-start guides to time-series analysis.

As a final note, it is worth reiterating that one of the opportunities provided by big data and data analytic approaches is that many such datasets and tools are free. There are also emerging systems to support free (or with very cheap initial cost) cyberinfrastructure to support access to datasets and processing power across academic institutes and communities. A leading example is the National Research Platform (Smarr et al., 2018; National Research Platform, 2024), an NSF-supported initiative providing pooled resources across dozens of institutes across the United States and a growing number of partner institutes in other countries. As not only data but also tools to process and analyze those data become more broadly and evenly distributed, we anticipate the emergence of additional opportunities to engage with historically underrepresented communities for whom research may have previously been inaccessible due to cost barriers associated with clinical or laboratory research. As data-enabled opportunities for new knowledge grow, collaborations between circadian biologists and data scientists will accelerate the rate at which such opportunities can be realized.

## Supplemental Material

sj-jpg-1-jbr-10.1177_07487304241310923 – Supplemental material for Augmenting Circadian Biology Research With Data Science Supplemental material, sj-jpg-1-jbr-10.1177_07487304241310923 for Augmenting Circadian Biology Research With Data Science by Severine Soltani, Jamison H. Burks and Benjamin L. Smarr in Journal of Biological Rhythms
