# Supplementary figures and images for: Augmenting Circadian Biology Research With Data Science
Source: J Biol Rhythms. 2025 Jan 29;40(2):143–70. doi: 10.1177/07487304241310923 (PMC11915776; doi:10.1177/07487304241310923)

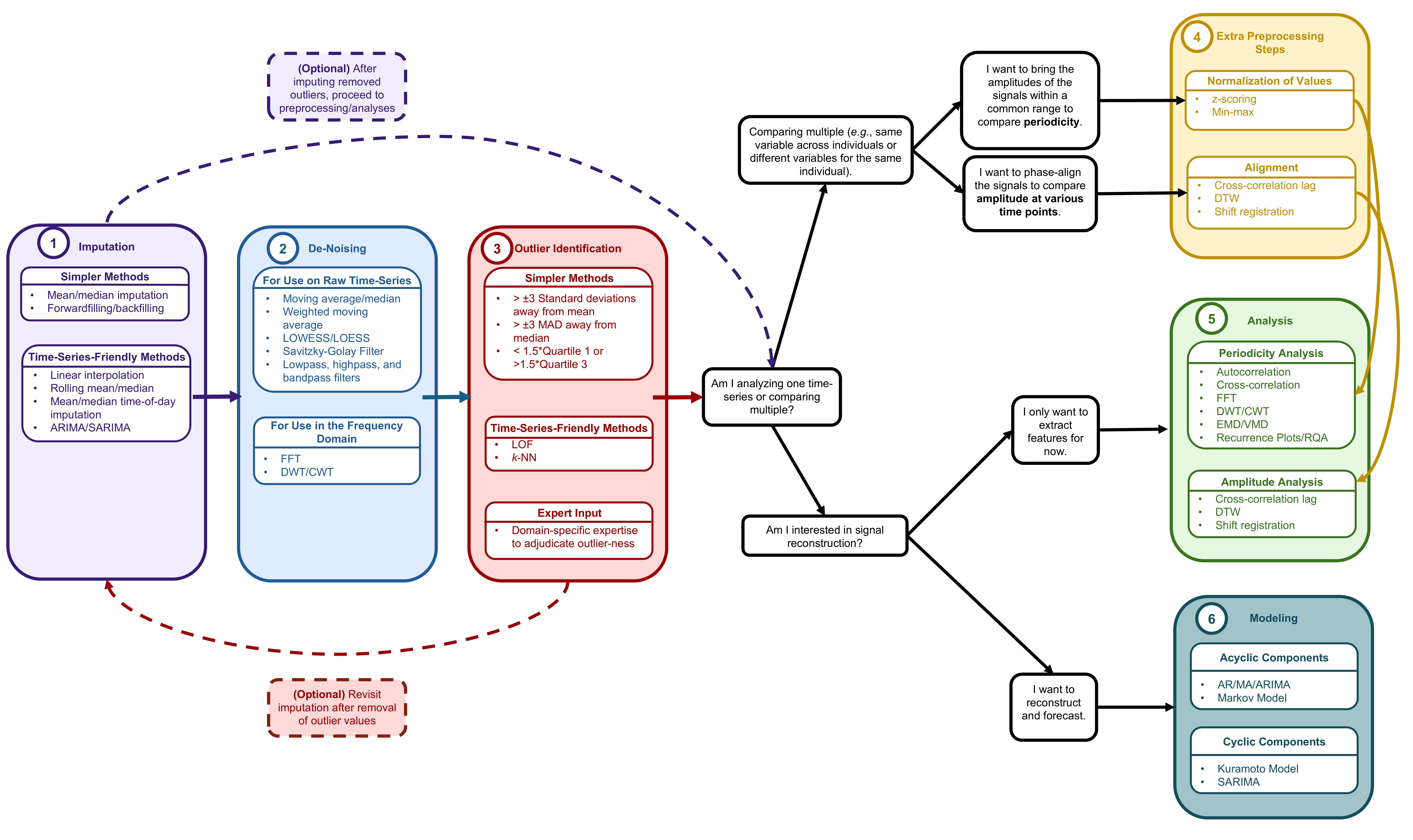

Supplement: sj-jpg-1-jbr-10.1177_07487304241310923 – Supplemental material for Augmenting Circadian Biology Research With Data Science [file sj-jpg-1-jbr-10.1177_07487304241310923.jpg]
